# The effects of ECMO on neurological function recovery of critical patients: A double-edged sword

**DOI:** 10.3389/fmed.2023.1117214

**Published:** 2023-03-30

**Authors:** Jinxia Cai, Halidan Abudou, Yuansen Chen, Haiwang Wang, Yiping Wang, Wenli Li, Duo Li, Yanxiang Niu, Xin Chen, Yanqing Liu, Yongmao Li, Ziquan Liu, Xiangyan Meng, Haojun Fan

**Affiliations:** ^1^Institute of Disaster and Emergency Medicine, Tianjin University, Tianjin, China; ^2^Wenzhou Safety (Emergency) Institute, Tianjin University, Wenzhou, China

**Keywords:** extracorporeal membrane oxygenation, brain, neuroprotection, neurologic complications, cardiac arrest

## Abstract

Extracorporeal membrane oxygenation (ECMO) played an important role in the treatment of patients with critical care such as cardiac arrest (CA) and acute respiratory distress syndrome. ECMO is gradually showing its advantages in terms of speed and effectiveness of circulatory support, as it provides adequate cerebral blood flow (CBF) to the patient and ensures the perfusion of organs. ECMO enhances patient survival and improves their neurological prognosis. However, ECMO-related brain complications are also important because of the high risk of death and the associated poor outcomes. We summarized the reported complications related to ECMO for patients with CA, such as north–south syndrome, hypoxic–ischemic brain injury, cerebral ischemia–reperfusion injury, impaired intracranial vascular autoregulation, embolic stroke, intracranial hemorrhage, and brain death. The exact mechanism of ECMO on the role of brain function is unclear. Here we review the pathophysiological mechanisms associated with ECMO in the protection of neurologic function in recent years, as well as the ECMO-related complications in brain and the means to improve it, to provide ideas for the treatment of brain function protection in CA patients.

## 1. Introduction

Extracorporeal membrane oxygenation (ECMO) is used clinically as a partial or complete replacement for cardiopulmonary function, providing effective respiratory/circulatory support, and is becoming an essential adjunctive support technique in clinical care (1). Since 2009, the use of ECMO has increased globally, from 3,262 cases to 20,317 cases in 2021, indicating a 623% or sixfold increase in use (2). In terms of indications, ECMO was mainly used to provide respiratory support (51% of cases), followed by cardiac support (37%) and assistance with extracorporeal cardiopulmonary resuscitation (ECPR) (12%). Especially in ECPR, ECMO is initiated emergently on patients who have had a cardiac arrest (CA) and on whom conventional cardiopulmonary resuscitation (CCPR) has failed. As well as the outbreak of COVID-19 in 2019, ECMO has played an indispensable role in the treatment of novel coronavirus pneumonia (3–5). ECMO is gradually showing its advantages in terms of speed and effectiveness of circulatory support (6–8), and its trend as acute cardiopulmonary life support is gaining ground worldwide (9–11).

ECMO has gradually drawn attention in the field of neuroprotection, which could remarkably increase long-term survival and improve the prognosis of neurological function to improve the life quality of critical care patients. In 2020, Yannopoulos et al. (12) reported that early application of ECMO improved survival from 7 to 43% in patients with “refractory cardiac arrest” compared with standard advanced cardiac life support (ACLS). ECMO significantly preserves neurological function and improves functional status scores after 6 months for survivors (12). Notably, this significant increase in survival and improvement in neurological function is inextricably linked to the possible neuroprotective function of ECMO. However, some studies have also shown that the use of ECMO may result in several brain-related complications due to improper cannulation or others. These complications include stroke, hypoxic–ischemic brain injury, seizures, intracranial hemorrhage, cerebral embolism (13, 14).

Therefore, ECMO can be a lifesaving therapy in patients with refractory severe respiratory and/or cardiac failure and improve survival. However, ECMO remains associated with significant neurologic morbidity and mortality. Here we review the neuroprotection pathophysiological mechanism of ECMO, as well as the ECMO-related brain complications, and the means to improve it, to provide ideas for the treatment of brain function protection in CA patients.

## 2. The benefits of ECMO in neurological function of CA patients

**Figure 1:**
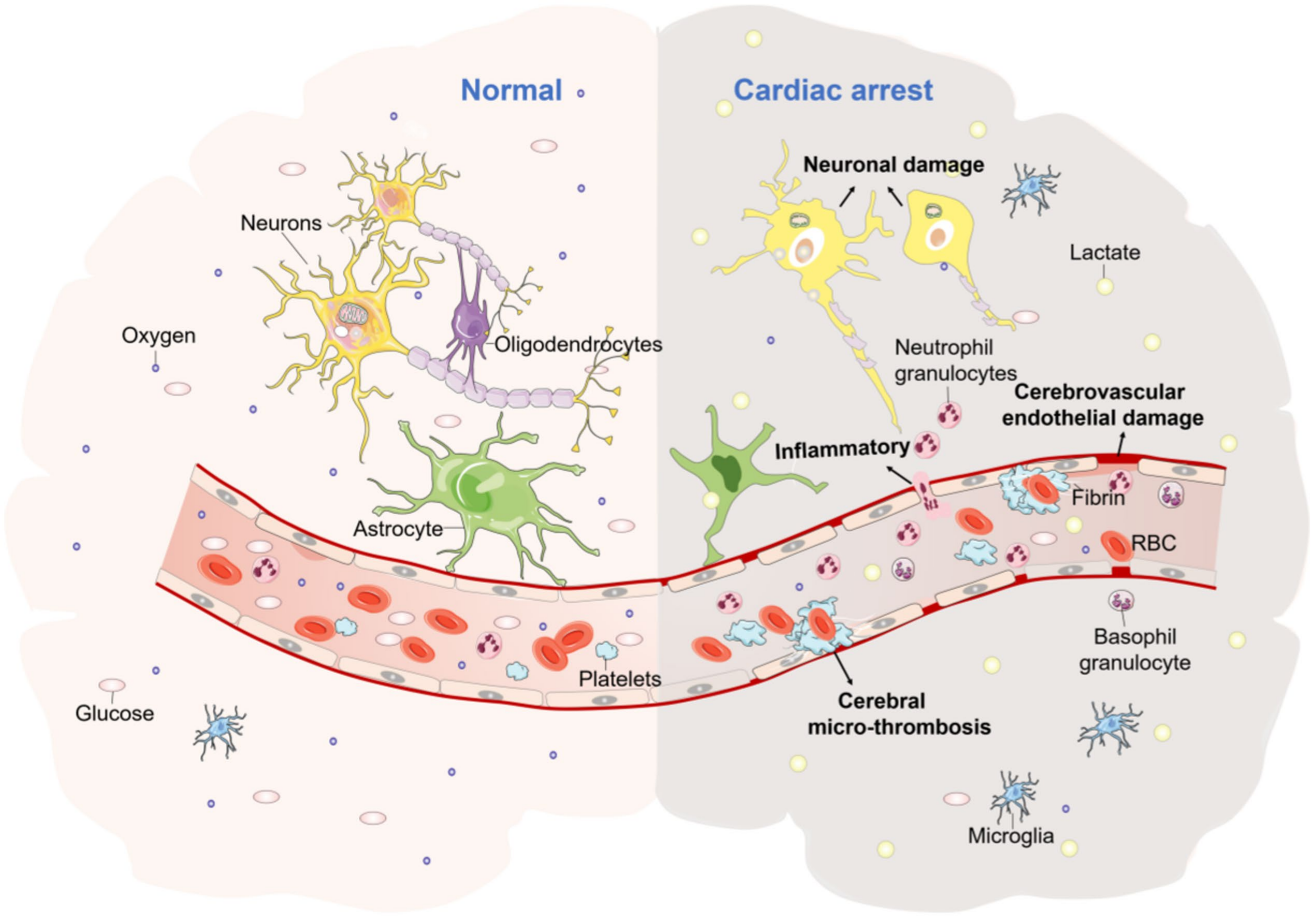
The pathophysiological mechanisms of CA brain injury. The picture represents the morphological changes in brain tissue due to ischaemia and hypoxia following CA, with increased lactic acid, cerebrovascular endothelial damage, increased inflammatory cells, cerebral micro-thrombosis, neuronal damage, dendritic morphological changes, and a range of other pathological processes occurring.

CA is a clinical emergency in which there is a sudden termination of the cardiac ejection, loss of large arterial pulsations and heart sounds, as well as severe ischemia and hypoxia in vital organs (e.g., the brain), resulting in the end of life. Post-cardiac arrest brain injury (PCABI) is a major cause of death and long-term disability (15). PCABI is caused by initial ischemia and subsequent reperfusion of the brain after resuscitation. CA causes a cessation of cardiac output and oxygen delivery to all vital organs (e.g., brain, etc.). Cerebral blood flow (CBF) is then stopped resulting in an immediate interruption of brain activity. Whereas brain tissue viability is largely dependent on a continuous supply of oxygen and energy substrates (i.e., glucose). Due to the lack of intrinsic energy stores, neurons are particularly vulnerable to ischemia, and cellular damage begins as soon as CBF is lacking. At the cellular level, ischemia leads to a cessation of aerobic metabolism and consequent depletion of the high-energy substrate adenosine triphosphate (ATP). This ultimately leads to a series of pathological processes such as neuronal damage and altered dendritic morphology (Figure 1) (15). The early introduction of ECMO can significantly improve the prognosis of PCABI and neurological function, mainly through improving brain histopathological damage and reducing the expression of brain injury biomarkers, reducing the inflammatory reaction, anti-Apoptosis of neuronal cells in the hippocampal CA1 region, antioxidant stress, and improving cerebral metabolism to play a protective role.

VA-ECMO is the main component of ECPR assistance after CA and can significantly improve the neurological prognosis of CA patients. Ouweneel et al. (16) showed that the use of ECMO for acute myocardial infarction combined with refractory cardiac arrest and cardiogenic shock was associated with improved survival and an increased favorable neurological prognosis in a 3,333 patient meta-analysis. Another systematic review of the literature on the neurological outcomes of 19 studies of in-hospital CA (IHCA) treated with ECPR found that the combined percentage of surviving patients with a good neurological prognosis (CPC score of 1 or 2) was 84% (17). Survival to hospital discharge and good neurological recovery in patients treated with ECPR vs. CCPR after out-of-hospital CA (OHCA) has also been evaluated, and a trend toward improved survival and good neurological outcomes with ECPR was also found (18). All of the above suggests that ECPR as a treatment for refractory CA is associated with a good neurological prognosis for the majority of patients.

## 3. Mechanisms of ECMO in preventing cerebral injury induced by CA

### 3.1. Reduction of brain injury biomarkers

Lower perfusion pressure after CA-CPR resulted in inhibiting brain metabolism and necrosis of neuron cells due to ischemia and hypoxia, releasing relevant brain injury biomarkers into the blood, such as NSE (neuron-specific enolase) and S100b (present in glial and membranous cells) (19). Serum biomarkers such as NSE and S100b have been identified as valid predictive markers of neuronal injury (20). The use of the biomarker S100b protein to determine non-traumatic, traumatic, and tumor-associated brain damage is accepted practice (21, 22). Equally, NSE is recognized as a valid prognostic biomarker for neurological outcomes after CA (23).

NSE is one of the enolases involved in the glycolysis pathway. NSE is most active in brain cells, detected in serum and cerebrospinal fluid, and has a half-life of 24–72 h. NSE consists of α, β, and γ subgroups, of which γ is specific to neuronal cells (24). The *European Resuscitation Council and European Society of Intensive Care Medicine Guidelines 2021* recommend a specific NSE cut-off value of 60 μg/L at 48 and/or 72 h after the return of spontaneous circulation (ROSC) to accurately predict outcomes after CA (25). Petermichl et al. (26), in a retrospective study of 69 patients, resuscitated after CA found NSE could be used to identify survivors 24 h after ECPR treatment, and neurological outcomes could be assessed as early as 48 h later. High serum NSE values at 48–72 h after CA support a poor prognosis of neurological prognosis, especially if repeated sampling results in consistently high values (27). Additionally, a study showed that the prognostic value of serum NSE in the group with severe blood–brain barrier disruption was more useful than that in the group without severe blood–brain barrier disruption (28), whereas a cut-off value of 34.6 μg/L in those with severe blood–brain barrier disruption had a sensitivity of 86.4% and a specificity of 100.0% (28).

A relative decrease in S100b during the first 24 h indicated a good neurological outcome, while an increase in S100b to base level in the first 24 h post-cannulation was associated with a worse neurological outcome. Good neurological outcome was associated with a continual decrease in S100b relative to the first value (measured within 12 h) (26). A delayed decrease in S100b or even an increase at 4 and 12 h after ECPR was associated with a poor neurological outcome (26). The sole decision about the neurological outcome according to CA and ECPR based on the S100b course does not be reliable, and the combination of NSE and S100b can improve the prognostic validity of neurological outcomes after CA and ECPR (26).

In addition, the prognostic biomarkers S100b, NSE, lactate, d-dimer, and interleukin 6 (IL-6) can be used as indicators of the neurological outcome when performing ECPR and TTM at the emergency scene (26). High serum lactate level was associated with poor survival and poor neurological outcome in CA patients treated with ECMO (29). Lactate and d-dimer were the earliest reliable prognostic markers for survival in ECPR. At 1 h after ECPR, non-survivors had a significantly increased lactate value compared to CPC 1–2 survivors (26). Other novel markers neurofilament light (NFL), tau, ubiquitin C-terminal hydrolase-L1 (UCH-L1), and glial fibrillary acidic protein (GFAP) are currently available as research-level tests (30). These biomarkers may show the highest predictive power and are believed to be an important addition to clinical tools in the near future.

### 3.2. Mitigation of the oxidative stress reaction

Ischemic–hypoxic damage of CA causes an increase of intracellular calcium, thus leading to glutamate release, arising calcium-dependent mitochondrial dysfunction, resulting in cellular energy failure and the release of pro-apoptotic proteins and reactive oxygen species (ROS), further leading to neuronal damage (15). However, the use of ECMO can reduce this oxidative stress damage in the brain.

Zhang et al. (31) found that ECMO could improve ATPase activity compared to CCPR in a porcine model of *CA.* It has also been found that ECPR treatment of CA in rats has a protective effect on neurons after CA, and the mechanism may be related to improved energy metabolism in the brain (32). A CA pig model study (33) found increased superoxide dismutase activity and decreased malondialdehyde and ROS in the ECMO group compared with CCPR. All of the above suggests ECMO can exert a neuroprotective effect by the mechanisms of antioxidant stress.

### 3.3. The alleviation of inflammatory reaction

ECMO alleviates the neuro-inflammatory response in brain tissue. Zhang et al. (31) in a porcine cardiac arrest model, found that IL-1, IL-6, TNF-α, and TGF-β levels were significantly lower in the ECMO group than in the CCPR group, while the difference in IL-10 levels between the two groups was not statistically significant. Pastuszko et al. (34) investigated the effect of ECMO on pro-inflammatory signaling in the striatum of piglets after CA and found that ECMO significantly reduced pro-inflammatory proteins (IL-12p40, IL-21, IL-15, IL-1α, and β, IL-8, MIP-1β, OPG, PIGF-2, RANTES, and TGF-β). Those reductions may lead to disturbances in neuronal metabolism and amplify inflammatory cell death (34).

However, other studies in a rat model of CA showed that the pro-inflammatory factor TNF-α was elevated in the CCPR group compared to the Sham group (*p* < 0.05), and the pro-inflammatory factor TNF-α was significantly elevated in the ECPR group (*p* < 0.001) and IL-6 was elevated in the ECPR group (*p* < 0.05), and the expression of pro-inflammatory factors was more severe in the ECPR group than in the CCPR group (32). Pastuszko et al. (34) found that ECMO could significantly reduce the expression of anti-inflammatory factors (ANG-1, FGF-21, IFN-α and β, IGF-2, IL-10, IL-13, IL-1ra, IL-22, IL-4, IL-6, NCAM-1, SCF, TGF-α, TIMP-1 and 2, VECF) in the striatum of piglets. A plausible explanation for the significantly increased plasma inflammatory cytokine (IL-6, TNF-α, CRP) and anti-inflammatory cytokine (IL-10) levels following ECMO treatment is the bacterial translocation following ECMO disconnection due to the contact of blood with the surface of the artificial line during ECMO (35), the non-pulsatile pulsation caused by the pump (36) and the non-laminar flow (37), which causes an inflammatory response activation of the immune response and its possible simultaneous activation of the anti-inflammatory response through stimulation of the vagus nerve (38). Therefore, ECMO may alleviate the neuro-inflammatory response in brain tissue after CA by restoring adequate blood flow promptly.

### 3.4. Anti-apoptosis of neuronal cells

Neurons in the central nervous system differ in their sensitivity to ischemia–reperfusion injury (I/R). The striatum is involved in all aspects of motor behavior control and behavioral plasticity and is highly sensitive to hypoxia/ischemic injury in different brain regions (34). Neurons in the CA1 region of the hippocampus are most sensitive to I/R injury in the central nervous system (39).

ECMO reduced apoptosis in the striatum of the brain and the CA1 region of the hippocampus after *CA.* Xue compared the efficacy of ECPR and CCPR for CA rats and found that the expression of the apoptosis-regulating protein Bcl-2 and the Bcl-2/Bax ratio was reduced in the hippocampus of the ECPR group compared with the CA group (*p* < 0.05), suggesting that the neurons in the ECPR group are more resistant to apoptosis (32). Pastuszko et al. (34) investigated the effect of ECMO on pro-apoptotic signaling in the striatum of piglets after CA and found that the pro-apoptotic proteins (Bax, cytoC, IGFBP-6, TNF-β, and TRAIR 1 and 3) were significantly lower in the ECMO group compared to the CA group and the Bcl-2 to Bax ratio was increased by 30% in the ECMO group. But the expression of anti-apoptotic proteins (Bcl-2, Bcl-w, HSP27, HSP60, HSP70, IGFBP-1, IGFBP-3, Livin, Survivin) between the CA and ECMO groups are no different. Suggesting a protective effect of ECMO against striatum cell injury. In a piglet model of CA, ECMO significantly reduced the levels of pro-apoptotic proteins without altering the levels of anti-apoptotic proteins (34).

### 3.5. Improvement of cerebral metabolic function

The lack of energy after CA ischemia causes a series of subsequent neuronal injuries. As the increased release, reduced re-intake, and massive release of excitatory amino acids cause neurotoxic effects, arousing a subsequent series of neuronal injuries. The use of ECMO can ameliorate this injury.

Schober et al. (40) in the 8 min CA rat model, found that ECMO application elevated central nervous blood glucose levels and reduced lactate/pyruvate (L/P) ratio and glutamate levels. Pingfei (32) also in a CA rat model found that aspartic acid, glycine, and the lactate/pyruvate ratio were significantly elevated in both the CCPR and ECPR groups compared with the sham group. But both aspartic acid and glycine were decreased in the ECPR group compared to the CCPR group (32). However, glutamate concentrations were not significantly elevated in the ECPR and CCPR groups, which may be related to the transfer of astrocytes through the glutamate-glutamine cycle during the acute phase of injury (32). Another result of the ECPR low-flow model developed by Putzer et al. (41) showed that the use of epinephrine improved cerebral perfusion pressure, local CBF, cerebral oxygenation, cerebral metabolism and that the target means arterial pressure (MAP) of 60 mmHg during ECMO can improved metabolism. All the above suggest that ECMO can improve cerebral metabolic function.

**Figure 2:**
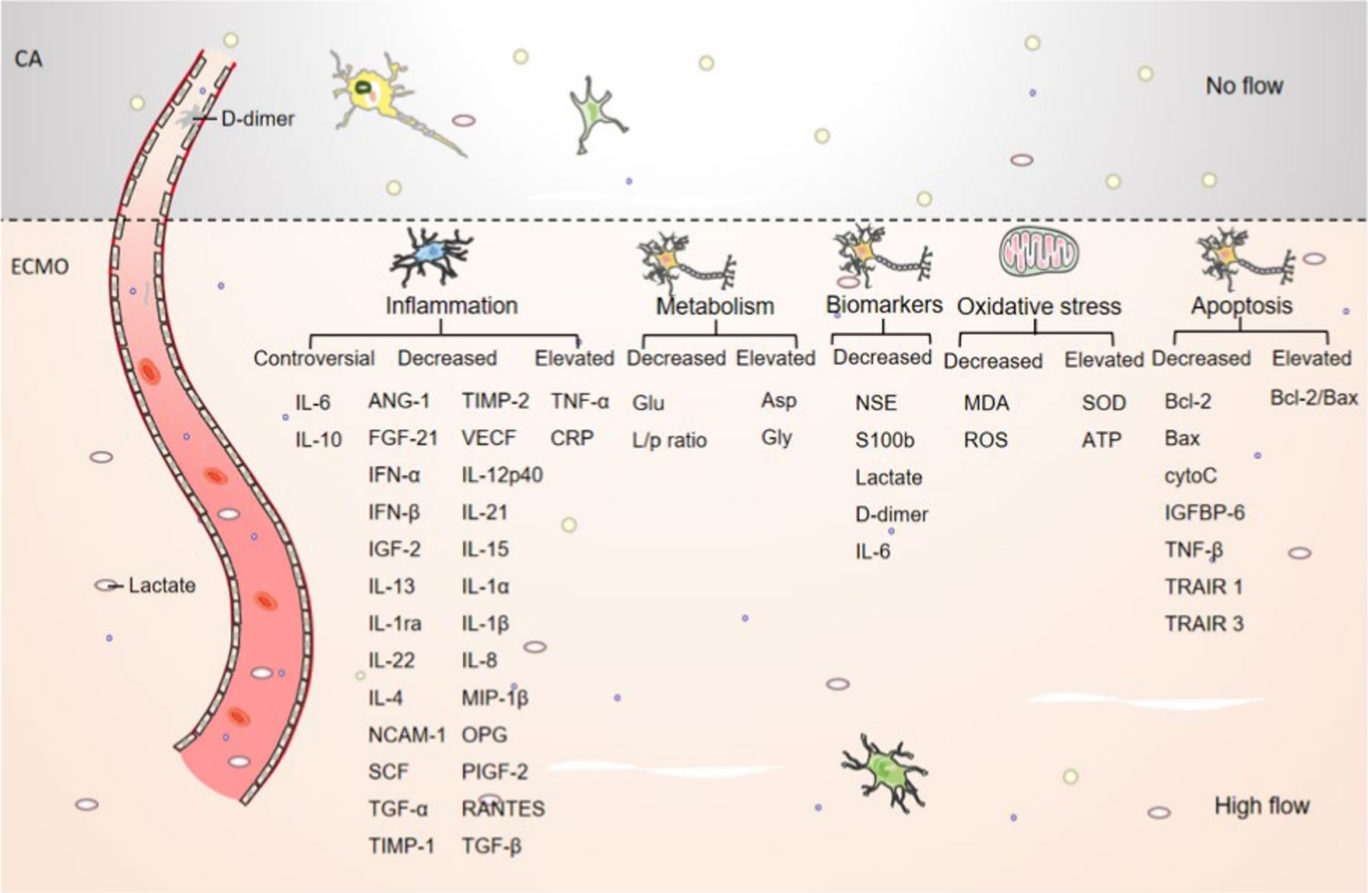
Schematic representation of the cerebral effects of ECMO. The number of brain neurons and nissls was elevated after ECMO resuscitation compared to *CA.* The number of lactate and d-dimer was decreased after ECMO resuscitation compared to *CA.* and other inflammatory, metabolic, necrotic, oxidative stress, apoptotic and other cytokine trends are shown in picture.

In conclusion, the mechanism of the cerebral protective effect of ECMO may be through alleviating brain cell necrosis, improving energy metabolism in the brain, inhibiting neuronal cell apoptosis, brain tissue inflammatory response, and anti-oxidant stress, thereby effectively improving various physiological functions and brain injury in *CA.* The mechanism of cerebral protective effect after ECMO treatment is shown in Figure 2.

## 4. Neurological complications of ECMO

Although ECMO offers a promising approach to the treatment of critically ill patients, there is a high incidence of neurological complications during ECMO treatment, due to high oxygen consumption, low oxygen reserve capacity, and poor tolerance to hypoxia of brain tissue being the organs most vulnerable to damage (15). Despite this technical success, ECMO is associated with high morbidity and mortality rates due to the invasive nature of the treatment and the technical complexity of the system (42). Complications associated with ECMO can be considered in two categories; patient-related and technical complications or failures. These complications include but are not limited to hypoxic–ischemic brain injury, cerebral I/R injury and impaired intracranial vascular autoregulation, cerebral embolism, and intracranial hemorrhage (14, 42, 43).

### 4.1. Hypoxic–ischemic brain injury

The primary conditions such as the no and low blood flow state after CA and during CPR could cause initial hypoxic–ischemic damage to the brain (44), and inappropriate ECMO cannulation can exacerbate this pathological damage.

Migdady et al. (45) performed a comprehensive systematic review and meta-analysis to identify specific neurologic complications of ECMO when it is deployed for purposes of refractory *CA.* They found a high frequency of ECPR-associated brain injury (27%), the most common of which was a hypoxic–ischemic brain injury. The mechanism of hypoxic–ischemic brain injury after ECPR is complex, as it relates to the underlying condition that prompts ECMO treatment (i.e., CA) as well as complications associated with ECMO use (i.e., Harlequin syndrome).

Harlequin Syndrome (also known as North–South Syndrome) is a complication of V-A ECMO that can occur when the left ventricular function starts to recover. When VA-ECMO is using femoral artery-venous cannulation, the cardiac output consists of blood from both the ECMO system and the patient’s left heart. If the patient has inadequate lung function or ventilator support, the blood ejected from the left heart has a low oxygen content. The ECMO cannula retrograde input of oxygenated blood from the femoral artery is competition for deoxygenated blood from the natural circulation (46–48). Which results in inadequate coronary and head oxygenation and severe bilateral cerebral hypoxia (44, 49). In addition, the clinical experience shows that the reduced “protective” lung ventilation used in ECMO patients may further contribute to varying degrees of hypoxemia (50). All of those may cause hypoperfusion of brain tissue and ischemia, leading to ischemic and hypoxic brain damage.

### 4.2. Cerebral IR injury

CA patients are in a state of severe ischemia, and although the use of ECMO offers hope for life, it is undeniable that ECMO may also increase the risk of organ reperfusion injury, particularly in the brain, with potentially more serious consequences for the patient. The mechanism of I/R injury in the brain is related to the activation of ROS and the production of free radicals after reperfusion, among others. This because the adequate blood supply leads to the formation of ROS, altered microvascular blood flow, endothelial disruption, and increased permeability of the blood–brain barrier, which may develop into a “sepsis-like” syndrome with a systemic cytokine response (45, 51–55). As well as CA patients treated with ECPR after ROSC usually accompanied with hyperoxia (usually defined as mild PaO_2_ > 100 or 120 mmHg and severe PaO_2_ > 300 mmHg) can also lead to acute cerebral IR injury. The underlying mechanism is that hyperoxia increases the production of ROS, which causes damage to lipid membranes, deoxyribonucleic acid and proteins (44), and neuronal damage. It also causes reactive vasoconstriction, leading to impaired cerebral microcirculation, and platelet dysfunction, which increases the risk of thrombosis and hemorrhage (56, 57). In addition, the de-cannulation of ECMO can also induce a systemic inflammatory response syndrome response, leading to further ROS production and increasing the likelihood of secondary acute brain injury (58).

### 4.3. Impaired autoregulation of intracranial blood vessels

Cerebral autoregulation is the ability to keep CBF almost constant for some range of changing the MAP. When VV-ECMO is used in patients with severe hypercapnia due to respiratory failure, a sudden drop in arterial blood carbon dioxide partial pressure levels leads to cerebral vasoconstriction and a dramatic reduction in CBF (59). The rapid transition from hypercapnia to a normal or hypercapnic state may lead to impaired cerebral self-regulation, further increasing the risk of complications such as cerebral ischemic injury (59, 60).

Another possible neurological risk factor specific to VA-ECMO is the loss of pulsatile systemic blood flow. Loss of pulsatile blood flow is a consequence of ECMO’s continuous blood flow system and is also associated with endothelial dysfunction, increased sympathetic tone, reduced local oxygen consumption, and increased systemic vascular resistance (36, 61, 62). In patients with impaired cerebral autoregulation, the use of potent sedatives during ECMO can reduce MAP due to peripheral vasodilation, and then lead to cerebral perfusion deficit (63). These may lead to intracranial vascular hyper- or hypo-responsiveness, which increases the risk of cerebral edema (64). In addition, several other factors such as irregular use of high doses of vasoactive drugs may also lead to impaired intracranial vascular autoregulation (45, 49).

### 4.4. Cerebral embolism or anticoagulation-related hemorrhage

Dysregulation of anticoagulation during ECMO can lead to intracranial hemorrhage or thrombosis. VA-ECMO has a high risk of embolic stroke because of the direct return of oxygenated blood to the aorta (usually the aorta or femoral artery), mostly due to solid and/or gaseous microemboli and thrombus formation in the catheter (65). In contrast, in VV-ECMO, the pulmonary circulation acts as a filter for emboli from the ECMO circuit (65, 66). In addition, the oxygenator membrane may be pro-thrombotic and requires systemic anticoagulation. These phenomena lead to thrombosis, hemolysis, and thrombocytopenia, increasing the risk of ischemic and hemorrhagic craniocerebral injury (44).

Factors that may lead to the risk of cerebral hemorrhage also include a systemic inflammatory response to ECMO circuits. Contact between blood and ECMO circuits can cause a systemic inflammatory response, including disruption of the blood–brain barrier and direct damage to neurons (43). Uptake of plasma proteins, activation of factor X, and thrombin production may further lead to an imbalance between the intrinsic procoagulant and anticoagulant regulatory systems, which increases the risk of abnormal bleeding and/or thrombosis (67). The triggered inflammatory cascade also leads to increased cytokine release, which in turn leads to thrombocytopenia and diffuse activation of the coagulation system, resulting in the depletion of blood clotting function (68, 69). In addition, the positive and negative pressures generated by the ECMO pump itself and within the circuit can lead to mechanical damage, dysfunction, and lysis of blood cells and platelets. As well as loss of von Willebrand factor, which may further predispose to bleeding (69, 70).

## 5. Methods to avoid or treat cerebral complications during ECMO

The cerebral complications during ECMO can be treated by treating the primary disease of the patient or by adjusting the mode of ECMO cannulation. ECMO can also combine with blood flow regulation, targeted blood pressure management, and EEG utilizing, as well as a combination of target temperature management (TTM), intra-aortic balloon pump (IABP), continuous renal replacement therapy (CRRT) and gas therapy [hydrogen (H_2_) and carbon monoxide (CO)] to achieve further improvements in brain function. This reduces the incidence of complications and brain damage and provides a solution for better brain protection in critically ill patients using ECMO.

### 5.1. Appropriately regulating ECMO ventilation/flow mode

Generally, the north–south syndrome, arising in VA-ECMO patients, is clinically addressed by increasing ECMO flow, ventilator-inspired oxygen concentration, and positive end-expiratory pressure, or by changing VA-ECMO to VAV-ECMO mode (49). VA-ECMO using central or axillary arterial cannulation may also be considered if severe differential hypoxia is present (46, 71, 72). If the patient has largely recovered cardiac function but not fully recovered respiratory function, he may be switched to VV-ECMO assistance to maintain his right upper extremity arterial oxygen saturation to 90–95% (49). For the impaired cerebral self-regulation produced by VV-ECMO patients, low gas flow may be adopted at the start of ECMO to avoid rapid correction of hypercapnia. At the initiation of either VA-ECMO or VV-ECMO, it would be prudent to limit the rate of CO_2_ reduction to avoid over-anticoagulation (59, 73). Because of the body’s pulmonary oxygenation in VA-ECMO mode, so intelligent regulation between ventilation and perfusion is very crucial.

### 5.2. TTM

TTM is the management of the body’s temperature to a target temperature to reduce organ tissue damage. The American Heart Association recommends a target temperature of 32–36°C for use in comatose adult patients after ROSC (27). The current advantage of ECMO is that it enables rapid cooling of the blood and maintain due to the heat exchangers (74), which has been demonstrated in an adult pig model within minutes (75, 76). Several studies have found that early TTM (subfreezing ~34.0°C) after ECMO can improve survival and neuroprognosis (77–79), as well as reduce cerebral injury (80, 81). Otherwise, another consideration for TTM in this population is that ECMO-related coagulation disorders may be exacerbated by lower temperature targets and the need for systemic anticoagulation.

Numerous clinical and animal studies have shown that hypothermia has an important neuroprotective effect on brain I/R injury after *CA.* Hypothermia can reduce CBF, reduce cerebral edema, decrease brain metabolism, reduce the production of ROS, reduce the release of excitatory amino acids and pro-inflammatory mediators, and inhibit apoptosis (82–84). TTM reduces endoplasmic reticulum stress-induced neuronal apoptosis, and changes in temperature within a certain sub-cold range do not diminish this protective effect (85, 86). In a study in CA rats, it was shown that both TTM alone and inhaled molecular H_2_ alone inhibited neuronal degeneration and microglia activation in vulnerable brain regions, with the combination of TTM and H_2_ inhalation being the most effective treatment (87).

### 5.3. Small molecule gases with anti-oxidative stress properties

I/R injury is an important cause of rapid, acute oxidative stress in post-cardiac arrest syndrome (PCAS), and ECMO administered with different concentrations of H_2_ can inhibit cerebral oxidative stress injury. H_2_ attenuates oxidative stress (87) leading to a variety of effects, including anti-inflammatory and anti-apoptotic responses through changes in gene expression (88), signal transduction (89, 90), and mitochondrial membrane potential (91, 92). H_2_ reacts with strong oxidants (e.g., -OH) in cells, but remains sufficiently mild to neither interfere with metabolic redox reactions nor signal ROS (92). Furthermore, H_2_ diffuses rapidly into tissues and cells, and it may also be suitable as a defense against acute oxidative stress induced by I/R injury (91, 93). Study shows that inhalation of therapeutic doses of H_2_ has no adverse effects on arterial oxygen saturation (SpO_2_) or hemodynamic parameters (including blood pressure, heart rate, and left ventricular pressure) (93). ECMO Administered with 2% H_2_ decreased mortality in ECPR-resuscitated CA rats and helped to restore electrical brain activity (94), the underlying mechanism may be related to the protective effect on endothelial injury (95). Therefore, ECMO administered with different concentrations of H_2_ can be used as an effective to avoid or treat cerebral oxidative stress injury.

Notably, ECMO combined with the endogenous gas-transmitter CO has attracted attention in reducing brain injury. In a porcine ECPR model, the addition of low doses of CO targeting 7–13% carboxyhemoglobin (CO-Hb) using a new *in vitro* CO delivery method reduced brain injury and improved neurological function (44). In addition, there are several gases (xenon, hydrogen sulfide, nitrogen monoxide, nitrogen dioxides), drugs, and, filter adsorption devices for ROS scavenging can also improve cerebral I/R injury, thus exerting a protective and reparative effect on neurological damage.

### 5.4. Other methods

The improvement of ECMO-related brain complications cannot be achieved without monitoring and early warning of problems of brain function. Several advanced devices (bedside transcranial doppler, CT, MRI, EEG, and near-infrared spectroscopy) can be used for monitoring and prognosis of brain function in critically ill patients (49, 96). Some blood markers [NSE, S100b (20), GFAP, lactate, among others] can also be used in the prediction of brain injury (26). All these standardized multimodal neuromonitoring methods, together with clinical neurological assessment and neurological consultation, may contribute to the early detection of brain injury associated with ECMO and thus allow early intervention.

The IABP and CRRT can also be used in combination with ECMO for the treatment of critically ill patients with different disease types. The IABP is a mechanical cardiac assist device that increases the blood and oxygen supply to the myocardium by inflating the balloon during diastole and deflating it before systole to decrease myocardial oxygen consumption (97). IABP was thought to provide left ventricle unloading and improve survival and good neurological outcomes in VA-ECMO (98). CRRT is a technique for the continuous, slow removal of water and solutes using extracorporeal circulation blood purification. CRRT was found to maintain hemodynamic stability, and high solute clearance, remove inflammatory mediators as well as safeguard the patient’s fluid balance and provide favorable conditions for total intravenous nutrition. Fang et al. (99) reported a 22-year-old prolonged CA patient who was resuscitated for over 75 min and treated with CRRT due to anuria, mixed acidosis, and hemodynamic instability. The results found that in hemodynamically unstable patients, administration of hypertonic saline solution and CRRT to maintain blood sodium levels within a therapeutic range of 148–150 mEq/L may mitigate the adverse effects of hypoxic brain injury.

**Figure 3:**
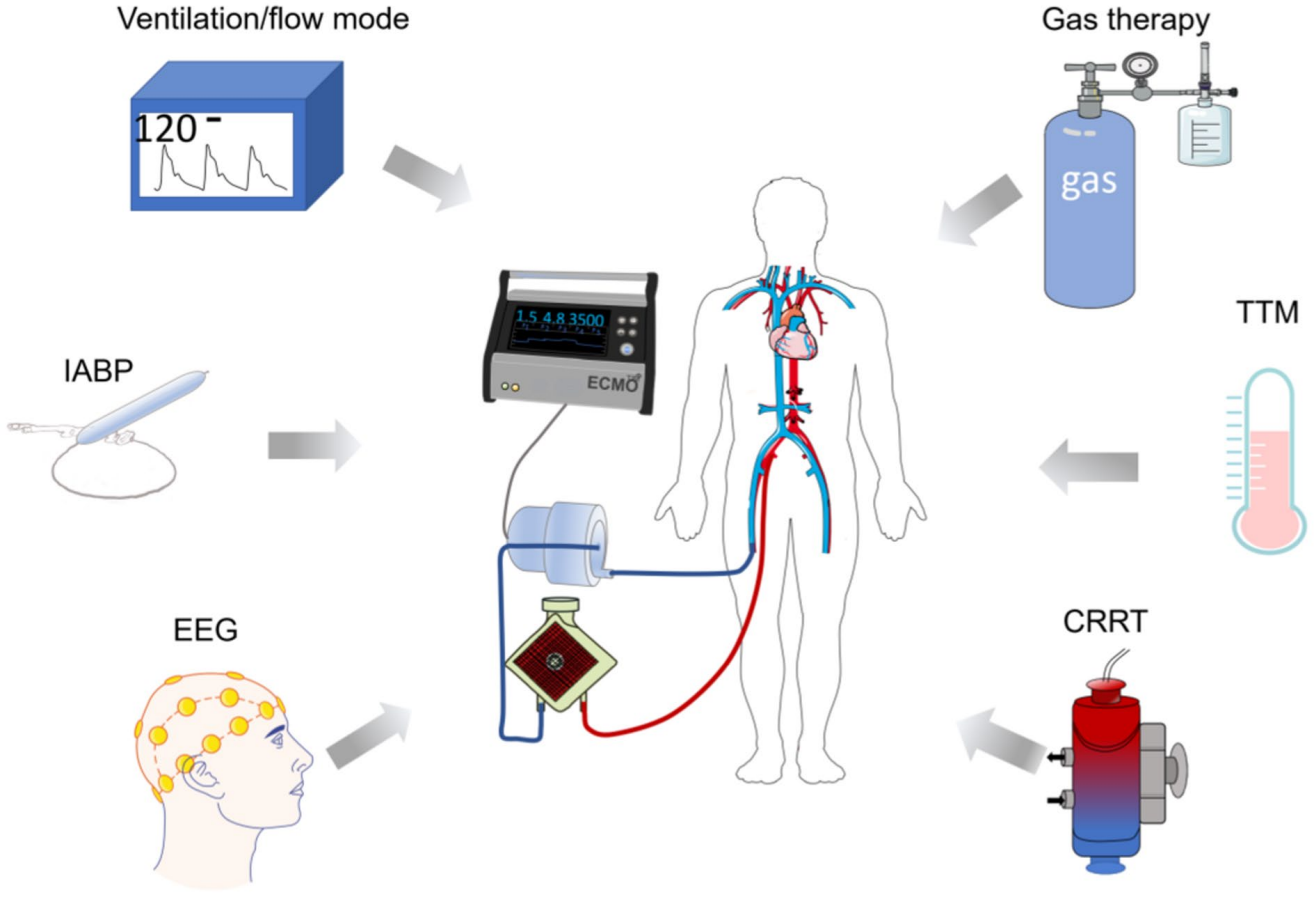
Diagram of ECMO in combination with other means of neuroprotection. The diagram indicates hemodynamic control, IABP, and EEG utilized during the treatment, respectively. Target temperature management, appropriate gas (H_2_, CO) interventions, and CRRT can assist in mitigating ECMO-related brain complications and provide ideas for neurological prognosis after ECMO use. TTM, target temperature management; CRRT, continuous renal replacement therapy; IABP, INTRA-aortic balloon pump.

Neurosurgical interventions are also available for complications such as cerebral embolism and cerebral hemorrhage. Neurosurgical interventions such as hematoma removal and/or decompression can be used for major intracerebral hemorrhage following the use of ECMO (100, 101). In patients with embolic stroke during ECMO, systemic thrombolysis should be avoided and catheter-directed thrombolysis and mechanical retrieval should be used (43, 102–104). Thromboelastography can also be used to accurately assess coagulation and reduce the risk of embolism and bleeding. Other tools such as flow regulation, targeted blood pressure management, and EEG utilized in ECMO are also beneficial for good neurological outcomes (97). Figure 3 summarizes the means of treatment for ECMO-related neurological complications.

## 6. Conclusion

As the use of ECMO has gradually increased, its indications have also expanded. ECMO can save time and give hope to critically ill patients, provide adequate cerebral perfusion and thus improve neurological prognosis. However, due to improper cannulation and other accidents, brain complications associated with ECMO often occur, including but not limited to hypoxic–ischemic brain injury, cerebral I/R injury and impaired intracranial vascular autoregulation, cerebral embolism, and intracranial hemorrhage. How to avoid complications during ECMO is a serious issue for every life support team.

The review is identifying the mechanisms of brain injury from previous studies and improves these mechanisms of injury, which may be effective for the prevention and treatment of ECMO-related brain complications. ECMO is convenient for a combination of blood flow regulation, targeted blood pressure management, and EEG utilization, as well as TTM, IABP, CRRT, and gas therapy (H_2_, CO) as modifiable parameters for the prevention and treatment of cerebral protection. Therefore, more research should be done on the prevention and treatment of ECMO-related brain complications.

In addition, the prognosis for brain injury is poor once it has occurred, so it is important to have multimodal monitoring and early warning of problems of brain function. The measurement of brain oxygen partial pressure will provide insight into changes in brain injury. For patients with brain injury who requires differentiated treatment, the treatment plans should be developed after individual assessment for different disease characteristics. Several advanced devices (bedside transcranial doppler, CT, MRI, EEG, and near-infrared spectroscopy) can be used for monitoring and prognosis of brain function in critically ill patients. Some blood markers (NSE, S100b, GFAP, lactate, among others) can also be used in the prediction of brain injury. All these standardized multimodal neuromonitoring methods, together with clinical neurological assessment and neurological consultation, may contribute to the early detection of brain injury associated with ECMO and thus allow early intervention. More research should be done on the monitoring and early warning of ECMO-related brain injury.

It is believed that with improved management, fewer complications, easier percutaneous manipulation, further experience of the ECMO team, implementation of effective means of multimodal monitoring of the brain, combined with optimized treatment of the appropriate complications, the future of ECMO for neuroprotection will have a bright prospect.

## Data availability statement

The data used to support the findings of this study are available from the corresponding author upon request.

## Author contributions

JC: conceptualization, data curation, writing—original draft, and writing—reviewing and editing. HA: methodology, investigation, and validation. YC: methodology, visualization, and software. HW, YaL, WL, DL, and YN: methodology and investigation. YW and XC: methodology and visualization. YoL: methodology and validation. ZL: validation, writing—reviewing and editing. XM: conceptualization, framework, and writing—reviewing and editing. HF: supervision, writing—reviewing and editing. All authors contributed to the article and approved the submitted version.

## Funding

This study was supported by the Scientific Research Translational Foundation of Wenzhou Safety (Emergency) Institute of Tianjin University (TJUWYY2022002).

## Conflict of interest

The authors declare that the research was conducted in the absence of any commercial or financial relationships that could be construed as a potential conflict of interest.

## Publisher’s note

All claims expressed in this article are solely those of the authors and do not necessarily represent those of their affiliated organizations, or those of the publisher, the editors and the reviewers. Any product that may be evaluated in this article, or claim that may be made by its manufacturer, is not guaranteed or endorsed by the publisher.
